# An Insect Prostaglandin E_2_ Synthase Acts in Immunity and Reproduction

**DOI:** 10.3389/fphys.2018.01231

**Published:** 2018-09-04

**Authors:** Shabbir Ahmed, David Stanley, Yonggyun Kim

**Affiliations:** ^1^Department of Plant Medicals, College of Natural Sciences, Andong National University, Andong, South Korea; ^2^Biological Control of Insects Research Laboratory, United States Department of Agriculture-Agricultural Research Service, Columbia, MO, United States

**Keywords:** eicosanoids, PGE_2_, PGES, immunity, reproduction, *Spodoptera exigua*

## Abstract

Eicosanoids, oxygenated metabolites of C20 polyunsaturated fatty acids (PUFAs), mediate fundamental physiological processes, including immune reactions and reproduction, in insects. Prostaglandins (PGs) make up one group of eicosanoids, of which PGE_2_ is a relatively well-known mediator in various insect taxa. While PG biosynthesis has been reported, the specific biosynthetic pathway for PGE_2_ is not known in insects. Here, we posed the hypothesis that *Se-mPGES2* mediates biosynthesis of physiologically active PGE_2_ through its cognate protein. To test this hypothesis, we interrogated a transcriptome of the lepidopteran insect, *Spodoptera exigua*, to identify a candidate PGE_2_ synthase (*Se-mPGES2*) and analyzed its sequence and expression. Its predicted amino acid sequence contains a consensus thioredoxin homology sequence (Cys-x-x-Cys) responsible for catalytic activity along with an N-terminal membrane-associated hydrophobic domain and C-terminal cytosolic domain. It also shares sequence homology (36.5%) and shares almost overlapping three dimensional structures with a membrane-bound human PGES2 (mPGES2). *Se-mPGES2* was expressed in all developmental stages with high peaks during the late larval instar and adult stages. Immune challenge significantly up-regulated its expression levels in hemocytes and fat body. Injecting double-stranded RNA (dsRNA) specific to *Se-mPGES2* significantly impaired two cellular immune responses, hemocyte-spreading behavior and nodule formation following bacterial challenge. Humoral immunity was also significantly suppressed, registered as reduced phenoloxidase activity and antimicrobial peptide expression levels. The suppressed immune responses were reversed following PGE_2_, but not arachidonic acid (AA), treatments. RNAi treatments also reduced the egg-laying behavior of females. Control females mated with the RNAi-treated males led to substantially reduced egg-laying behavior, which was also reversed following PGE_2_ injections into females. These results strongly bolster our hypothesis that *Se-mPGES2* acts in the biosynthesis of PGE_2_, a crucial biochemical signal mediating immune and reproductive physiology of *S. exigua*.

## Introduction

Eicosanoids are oxygenated metabolites of three C20 polyunsaturated fatty acids (PUFAs). Prostaglandin (PG) biosynthesis begins with hydrolysis of arachidonic acid (AA) from cellular phospholipids by a phospholipase A_2_ (PLA_2_). In vertebrates AA is then oxygenated by an enzyme with two catalytic sites, endoperoxide synthase/cyclooxygenase (COX). COXs are divided into a constitutively produced COX-1 and an inducible COX-2. PGs are potent lipid messengers involved in numerous homeostatic biological functions in mammals (Funk, 2001). PGs also act in various physiological processes such as reproduction, secretion, and immune responses in insects (Stanley and Kim, 2014).

Among various PGs, prostagladin E_2_ (PGE_2_) in mammals is a key mediator in inflammatory response and mediates other biological activities such as smooth muscle dilation and contraction (Smith et al., 1991), body temperature (Milton and Wendlandt, 1971), and the physiological sleep-wake cycle (Hayaishi, 1991). In invertebrates, PGE_2_ signaling is involved in immune responses and several aspects of oogenesis (Park et al., 2005; Spracklen et al., 2014).

Prostaglandin E synthase (PGES) catalyzes the isomerization of prostaglandin H_2_ (PGH_2_) to PGE_2_. In mammals and *Bombyx mori* (Yamamoto et al., 2013a), PGESs are homologs of Sigma class glutathione transferase (Kanaoka et al., 1997; Tanioka et al., 2000). X-ray crystallography of the enzyme and substrate indicates that there is an electron-sharing network at Asn95, Asp96, and Arg98, which is essential for glutathione isomerization (Yamamoto et al., 2013b). The electron-sharing network of PGES is assumed to act in the conversion of PGH_2_ into PGE_2_ (Sjögren et al., 2013).

In mammals, three PGESs have been identified, including one cytosolic, cPGES, and two microsomal, mPGES1 and mPGES2 (Gudis et al., 2005). cPGES is constitutively expressed and functionally coupled to COX-1 (Tanioka et al., 2000). mPGES1 is a perinuclear protein that is inducible and preferentially coupled to COX-2, causing a delayed PGE_2_ release response (Murakami et al., 2000). mPGES2 is synthesized as a Golgi membrane-associated protein and subsequent proteolytic cleavage of the N-terminal hydrophobic motif results in a cytosolic enzyme (Watanabe et al., 1997; Tanikawa et al., 2002). mPGES2 is constitutively expressed in various tissues and coupled to both COX-1 and COX-2 (Murakami et al., 2003). In arthropods, most PGESs are mPGES2s, except a shrimp, *Penaeus monodon*, which expresses three types of PGES (Wimuttisuk et al., 2013). However, phylogenetic analysis indicates that the arthropod mPGES2 sequences are distinctively clustered, separate from vertebrate orthologs (Eichner et al., 2015). Unlike vertebrate mPGES2s, the arthropod type exhibits a very low heme-binding affinity (Hansen et al., 2014).

Insect tissues produce a wide range of PGs. PGE_2_ is biosynthesized in the male reproductive tracts of the house cricket, *Acheta domesticus* (Destephano et al., 1974). Murtaugh and Denlinger (1982) detected PGE_2_ and PGF_2α_ in six insect species and reported their relative concentrations based on radioimmunoassay. Hemocyte and fat body (equivalent to vertebrate white blood cell and liver in function, respectively) preparations from *Manduca sexta* larvae biosynthesize several PGs (PGA_2_, PGE_2_, PGD_2_, and PGF_2α_) (Stanley-Samuelson and Ogg, 1994; Gadelhak et al., 1995).

In insects, PGE_2_ mediates various physiological processes including reproduction, fluid secretion, aging, and immunity (Stanley and Kim, 2011, 2014). Egg-laying behavior of *A. domesticus* and other crickets is stimulated by PGE_2_ (Stanley-Samuelson et al., 1986; Stanley, 2000). PGE_2_ also mediates events in egg development in some species including *Rhodnius prolixus* (de Medeiros et al., 2009). PGE_2_ plays major roles in modulating fluid secretion in Malpighian tubules, rectum and salivary glands. Treating hindgut (especially, rectal sac) of *Locusta migratoria* with PGE_2_ led to dose-dependent increases in fluid reabsorption (Radallah et al., 1995). Salivary gland fluid secretion in the blowfly, *Calliphora erythrocephala*, is negatively influenced by PGE_1_ by antagonizing a stimulating activity of serotonin (Dalton, 1977). Inhibiting PG biosynthesis with pharmaceutical inhibitors specifically suppressed fluid secretion in Malpighian tubules of a mosquito, *Aedes aegypti* and a forest ant, *Formica polyctena* (Petzel and Stanley-Samuelson, 1992; Van Kerkhove et al., 1995). Subsequent radiohistochemistry showed that PGE_2_ is localized in principal, but not in stellate, cells of *A. aegypti* Malpighian tubules (Petzel et al., 1993).

PGE_2_ also mediates various immune responses in insects (Stanley and Kim, 2011). Insect immunity is innate and triggered by sequential events initiated by recognition of nonself (Lemaitre and Hoffmann, 2007). Upon microbial pathogen infection, pattern recognition proteins recognize specific pathogen molecular patterns and activate cellular and humoral immune responses via immune mediators (Kurata, 2014). Various molecular immune mediators propagate the recognition signal to nearby immune-associated tissues such as hemocytes and fat body (Gillespie et al., 1997). Cross-talks between immune mediators use eicosanoids as the downstream signal (Sadekuzzaman et al., 2018). Especially, PGE_2_ mediates various immune responses including mobilization of sessile hemocytes (Park and Kim, 2000), hemocyte-spreading behavior (Srikanth et al., 2011), hemocyte nodulation, activation of prophenoloxidase (Shrestha and Kim, 2008), and induction of antimicrobial peptide gene expression (Yajima et al., 2003; Shrestha and Kim, 2007).

Unlike mammals, insects evolved another mechanism of PGE_2_ biosynthesis (Kim et al., 2018). First, PLA_2_ may catalyze the release of PUFAs other than AA. Though different PLA_2_s have been identified in insects, their phospholipids (PLs) have a very small amounts of AA, sometimes detectable only as trace, or catalytic, amounts (Stanley-Samuelson and Dadd, 1983; Kim et al., 2016). PLA_2_ may release C18 PUFAs from PLs, which may be converted into AA via elongation/desaturation pathways analogous to the mammalian counterparts (Stanley, 2000; Kim et al., 2018). Second, insects do not have typical mammalian COXs, but use a special peroxidase (=peroxinectin) to oxygenate AA into PGH_2_ (Park et al., 2014). Third, the synthesized PGH_2_ is likely to be isomerized into PGE_2_ by mPGES2 because other cellular PGES or mPGES1 are not identified in insect genomes. Two amphipod mPGES2s produce PGE_2_ from PGH_2_ (Hansen et al., 2014), from which we posed the hypothesis that *Se-mPGES2* catalyzes biosynthesis of physiologically active PGE_2_ through its cognate protein. Here, we report on the outcomes of experiments designed to test our hypothesis.

## Materials and Methods

### Insect Rearing and Bacterial Culture

*Spodoptera exigua* larvae used in this study were originated from Welsh onion field populations in Andong, Korea and were maintained in a laboratory for more than 20 years. The larvae were reared on an artificial diet (Goh et al., 1990) at 25 ± 1°C while adults were fed 10% sucrose solution. Under the diet condition, larvae developed for about 13 days from first instar (L1) to fifth instar (L5) before pupation. *Escherichia coli* Top10 (Invitrogen, Carlsbad, CA, United States) was cultured in Luria-Bertani (LB) medium (Becton, Dickinson & Co., Franklin Lakes, NJ, United States) overnight at 37°C with shaking at 200 rpm. For immune challenge, the bacteria were heat-killed at 95°C for 10 min and the bacterial cells were counted on a hemocytometer (Neubauer improved bright-line, Cat. No. 0640010, Superior Marienfeld, Germany) under a phase contrast microscope (BX41, Olympus, Tokyo, Japan). Heat-killing the bacteria was confirmed by growth failure after plating the treated bacteria on an LB plate and culturing at 28°C for 48 h. Bacterial suspension was diluted with sterilized and deionized distilled H_2_O for the preparation of treatment dose (5.4 × 10^4^ cells per μL).

### Chemicals

Arachidonic acid (AA: 5,8,11,14-eicosatetraenoic acid), prostaglandin E_2_ (PGE_2_: (5Z,11α,13E,15S)-11,15-dihydroxy-9-oxoprosta-5,13-dienoic acid), and dexamethasone (DEX: (11b,16a)-9-fluoro-11,17,21-trihydroxy-16-methylpregna-1,4-diene-3) were purchased from Sigma-Aldrich Korea (Seoul, Korea) and dissolved in dimethyl sulfoxide (DMSO). L-3,4-Dihydroxyphenylalanine (DOPA) was also purchased from Sigma-Aldrich Korea and dissolved in 10 mM with 100 mM phosphate-buffered saline (pH 7.4). Anticoagulant buffer (ACB) was prepared with 186 mM NaCl, 17 mM Na_2_EDTA, and 41 mM citric acid and then adjusted to pH 4.5 with HCl.

### Bioinformatics and Sequence Analysis

A *S. exigua* PGES2 sequence (*Se-mPGES2*) was obtained from the Transcriptome Shotgun Assembly (TSA) database deposited at GenBank^1^ using BlastN. The resulting sequence was subjected to open reading frame (ORF) analysis and its predicted amino acid sequence using Lasergene EditSeq program (Ver. 7.1, DNASTAR, Madison, WI, United States). Its ORF sequence was deposited at GenBank with the accession number of MG596301. Phylogenetic and domain analyses were performed using MEGA6 and ClustalW programs from EMBL-EBI^2^. Bootstrapping values were obtained with 1,000 repetitions to support branching and clustering. Protein domains were predicted using Pfam^3^ and Prosite^4^. Swiss-PDB Viewer^5^ and UCSF Chimera^6^ were used for protein motif and superimposition analysis.

### RNA Extraction and RT-PCR

Total RNAs were extracted from selected developmental stages using ∼500 eggs, 30 individuals for L1 or L2, 10 individuals for L3 or L4, and one individual for L5 for an experimental unit. To extract total RNAs from different tissues of L5 larvae, 3 days old L5 (L5D3) larvae were dissected in PBS. By cutting prolegs, hemolymph was collected and the remaining body was used to isolate fat body, midgut, and epidermis. The collected hemolymph in ACB was centrifuged at 800 × *g* for 3 min. The resulting hemocyte pellet was used to extract total RNA with Trizol reagent (Invitrogen, Carlsbad, CA, United States) according to manufacturer’s instruction. After DNase treatment, 1 μg of total RNA was used to prepare first-strand cDNA synthesized by RT-Premix oligo-dT (5′-CCAGTGAGCAGAGTGACGAGGACTCGAGCTCAAGCT(16)-3′, Intron Biotechnology, Seoul, Korea) in a reaction volume of 20 μL. The synthesized single-stranded cDNA was used as a template for PCR amplification with 35 rounds of a temperature cycle (95°C for 1 min, 52°C for 1 min, and 72°C for 1 min) after an initial heat treatment step at 95°C for 5 min with gene-specific primers (**Supplementary Table S1**). The PCR products were separated on 1% agarose gel under 100 V and subsequently stained with ethidium bromide. RT-qPCRs were performed with a qPCR instrument (CFX Connect^TM^ Real-Time PCR Detection System, Bio-Rad, Hercules, CA, United States) using SYBR^®^Green Realtime PCR Master Mix (Toyobo, Osaka, Japan) according to the general guideline suggested by Bustin et al. (2009). A ribosomal protein, RL32, gene was used as a stably-expressed reference gene (Park et al., 2015) for qPCR with gene-specific primers (**Supplementary Table S1**). Each cycle was scanned by measuring fluorescence intensity to quantify the PCR products. After the PCR reactions, melting curve analyses was performed from 60 to 95°C to ensure consistency and specificity of the amplified products. Each treatment was replicated three times using independent RNA collections. Quantitative analysis of gene expression was done using the comparative CT (2^−ΔΔCT^) method (Livak and Schmittgen, 2001).

### RNA Interference (RNAi) of Se-mPGES2 Expression

Template DNA was amplified with gene-specific primers (**Supplementary Table S1**) containing a T7 promoter sequence (5′-TAATACGACTCACTATAGGGAGA-3′) at the 5′ end. The resulting PCR product was used to *in vitro* synthesize double-stranded RNA (dsRNA) encoding *Se-mPGES2* (dsPGES2) using T7 RNA polymerase with NTP mixture at 37°C for 3 h. dsPGES2 was mixed with a transfection reagent Metafectene PRO (Biontex, Plannegg, Germany) in 1:1 (v/v) ratio and then incubated at 25°C for 30 min to form liposomes to increase RNAi efficiency. One mg of dsPGES2 was injected into 3 days old L4 (L4D3) using a microsyringe (Hamilton, Reno, Nevada, United States) equipped with a 26 gauge needle. The RNAi efficiency was determined by RT-qPCR against *Se-mPGES2* expression at 24 and 48 h post-injection (PI). Each treatment was replicated three times using independent RNA preparations.

### Nodule Formation Assay

Hemocytic nodules are formed as a cellular immune response of *S. exigua* in response to bacterial challenge (Park and Kim, 2000). This study used the heat-killed *E. coli* (5.4 × 10^4^ cells) for the immune challenge by injecting them through an abdominal proleg of L5D3 larvae, then incubating the larvae for 8 h PI at 25°C. The treated larvae were dissected on the dorsal side and the melanized nodules on its gut and fat body were initially counted under a stereoscopic microscope (Stemi SV11, Zeiss, Jena, Germany) at 50× magnification. After the alimentary canal was removed, nodules in the previously unexposed areas and remaining internal tissues were then counted and added to the initial count. Each treatment consisted of 10 test larvae. For RNAi experiment, at 24 h PI, the treated larvae were used in immune challenge. Each treatment used 10 larvae. A viral gene, *CpBV-ORF302*, was used as the negative RNAi control (Park and Kim, 2010).

### Immunofluorescence Assay for Hemocyte-Spreading Behavior

Total hemolymph (∼150 μL) from five L5 individuals was collected from a larval proleg into 850 μL of ACB and incubated on ice for 30 min. After centrifugation at 800 × *g* for 5 min, 700 μL of supernatant was discarded. Cell suspension was gently mixed with 700 μL of TC100 insect tissue culture medium (Welgene, Gyeongsan, Korea). Ten microliter of this hemocyte suspension was taken onto a glass coverslip and incubated in a wet chamber under darkness. Cells were then fixed with 4% paraformaldehyde for 10 min at room temperature (RT). After washing three times with PBS, cells were permeabilized with 0.2% Triton X-100 in PBS for 2 min at RT. Cells were washed once in PBS and blocked with 10% BSA in PBS for 10 min at RT. After washing once with PBS, cells were incubated with fluorescein isothiocyanate (FITC)-tagged phalloidin in PBS for 1 h at RT. After washing three times, cells were incubated with 4′,6-diamidino-2-phenylindole (DAPI, 1 μg/mL) (Thermo Scientific, Rockford, IL, United States) in PBS for nucleus staining. Finally, after washing twice in PBS, cells were observed under a fluorescence microscope (DM2500, Leica, Wetzlar, Germany) at 400× magnification. Hemocyte-spreading was determined by the extension of F-actin out of the original cell boundary.

### Phenoloxidase (PO) Enzyme Assay

Plasma PO activity was determined using DOPA as the substrate. Each L5D3 larva was injected with 5.4 × 10^4^ cells of heat-killed *E. coli*. At 8 h PI, 500 μL of hemolymph from ∼10 treated larvae were collected into 1.7 mL tube. Hemolymph was centrifuged at 4°C for 5 min at 800 × *g* to collect the plasma fraction supernatant. The total reaction volume (200 μL) consisted of 180 μL of 10 mM DOPA in PBS and 20 μL of the plasma sample. Absorbance was read at 495 nm using (VICTOR multi label Plate reader, PerkinElmer, Waltham, MA, United States). PO activity was expressed as ΔABS/min/μL plasma. Each treatment consisted of three biologically independent replicates.

### Egg-Laying Behavior

For analysis of egg-laying behavior, 5 days old male and female pupae were separated before adult emergence. PGE_2_ was injected to virgin females at 10 μg per adult on the day of adult emergence. Total numbers of eggs laid by virgin (without male) or mated females (with males in 1:1 sex ratio) were counted for 3 days after adult emergence. To determine whether *Se-mPGES2* acts in egg-laying behavior, RNAi was performed by injecting 1 mg of gene-specific dsRNA, and the viral gene *CpBV-ORF302* for controls, as described above. Each treatment consisted of three replicates, each with 10 females.

### Statistical Analysis

Data from all assays were analyzed by one-way ANOVA by PROC GLM for continuous variables using SAS program (SAS Institute Inc., 1989). The means were compared by least squared difference (LSD) test at Type I error = 0.05.

## Results

### Functional Domain Analysis of Se-mPGES2

**FIGURE 1:**
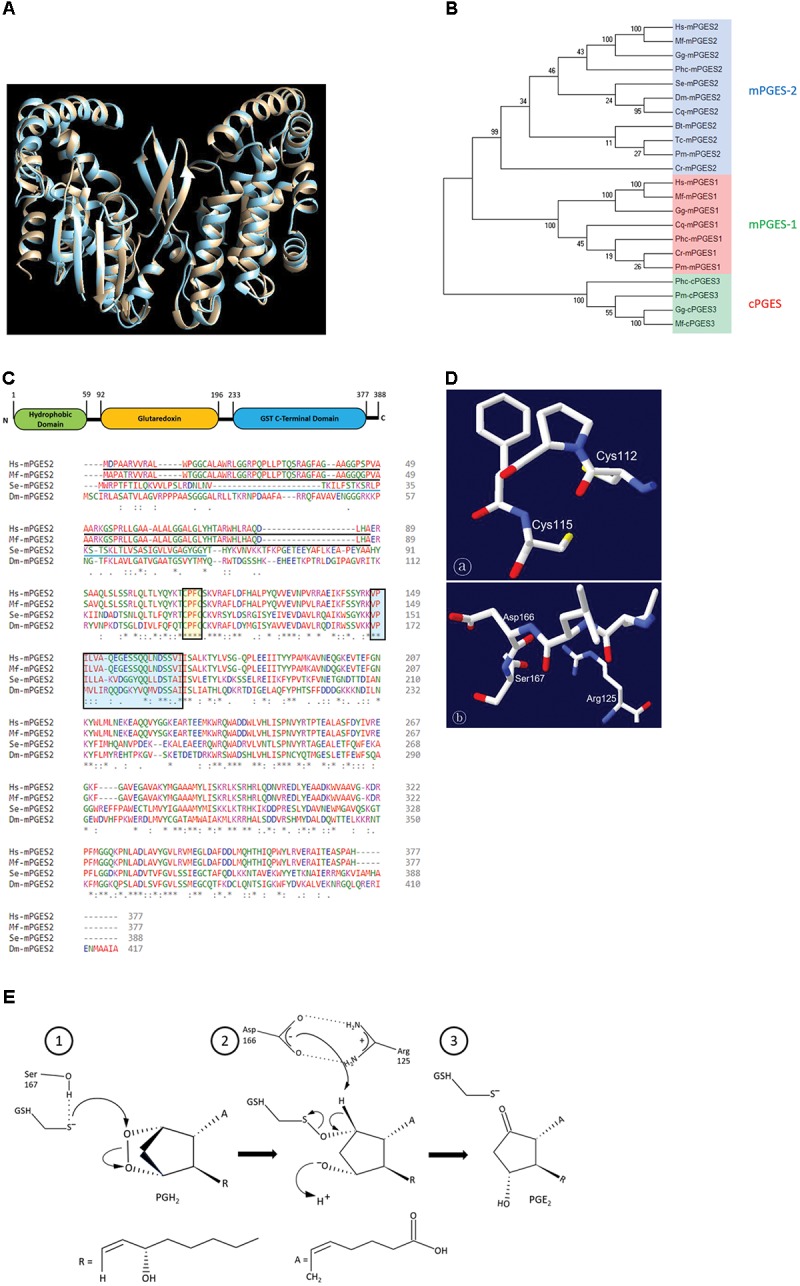
Molecular characteristics of a *S. exigua* PGES (*Se-mPGES2*). **(A)** Structural similarity of *Se-mPGES2* to a primate mPGES2. A superimposed view of *Macaca fascicularis* mPGES-2 crystal structure (gray) and the predicted three-dimensional structure of *Se-mPGES2* (blue). Superimposition analysis was performed by UCSF Chimera (https://www.cgl.ucsf.edu/chimera/). **(B)** A phylogenetic analysis of *Se-mPGES2*. The analysis was performed using MEGA6. Bootstrapping values were obtained with 1,000 repetitions to support branching and clustering. Amino acid sequences of selected mPGES2 genes were retrieved from GenBank: NP_004869.1 for *Homo sapiens* (Hs-mPGES1), ADK66307.1 for *Gallus gallus* (Gg-mPGES1), AAS89037.1 for *M. fascicularis* (Mf-mPGES-1), ACO11433.1 for *Caligus rogercresseyi* (Cr-mPGES1), AFJ11395.1 for *Penaeus monodon* (Pm-mPGES-1), XP_001863047.1 for *Culex quinquefasciatus* (Cq-mPGES1), EEB10983.1 for *Pediculus humanus corporis* (Phc-mPGES1), NP_079348.1 for *H. sapiens* (Hs-mPGES2), XP_415498.1 *G. gallus* (Gg-mPGES2), BAB01608.1 for *M. fascicularis* (Mf-mPGES2), ACO11658.1 for *C. rogercresseyi* (Cr-mPGES2), NP_524116.2 for *D. melanogaster* (Dm-mPGES-2), XP_002432321.1 for *P. humanus corporis* (Phc-mPGES2), XP_001868980.1 for *C. quinquefasciatus* (Cq-mPGES-2), XP_003403370.3 for *Bombus terrestris* (Bt-mPGES2), XP_973652.1 for *Tribolium castaneum* (Tc-mPGES2), AFJ11396.1 for *P. monodon* (Pm-mPGES2), Q90955.1 for *G. gallus* (Gg-cPGES3), AAS89038.1 for *M. fascicularis* (Mf-cPGES3), XP_002430923.1 for *P. humanus corporis* (Phc-cPGES3), and AFJ11394.1 for *P. monodon* (Pm-cPGES3). **(C)** Domain analysis of *Se-mPGES2*. The domains of *Se-mPGES2* were predicted using Pfam (http://pfam.xfam.org) and Prosite (https://prosite.expasy.org/). *Se-mPGES2* was aligned with the deduced amino acid sequences of *H. sapiens*, *M. fascicularis*, and *Drosophila melanogaster* mPGES2. Identical amino acids were marked with asterisks while similar amino acids were denoted with colons. The N-terminal hydrophobic domain was predicted and underlined with a solid line. The Cys-Pro-Phe-Cys motif and predicted GSH-binding motif were boxed. **(D)** A model structure of the Cys-Pro-Phe-Cys motif and GSH-binding motif. Swiss-PDB Viewer (http://spdv.vital-it.ch/) was used for detection of protein motifs and active sites. **(E)** A proposed mechanism of the catalytic activity of *Se-mPGES2* against isomerization of PGH_2_ into PGE_2_.

*Se-mPGES2* was predicted from a transcriptome (GenBank accession number: GARL01090824.1) by interrogation with a *B. mori* PGES2 sequence (GenBank accession number: XP_012548985.1) as a query. Its ORF consists of 1,167 bp encoding 388 amino acids. *Se-mPGES2* had 36.5, 36.1, and 40.0% amino acid sequence similarities with *Homo sapiens*, *Macaca fascicularis*, and *Drosophila melanogaster* mPGES2s, respectively. The predicted protein structure of *Se-mPGES2* was compared with the crystal structure of *M. fascicularis* mPGES2 which showed high similarity by superimposition (**Figure 1A**). Phylogenetic analysis shows three clusters of cPGES, mPGES1, and mPGES2, in which *Se-mPGES2* was included in the mPGES2 cluster (**Figure 1B**). Like mammalian mPGES2s, *Se-mPGES2* was comprised of an N-terminal membrane-associated hydrophobic domain and a cytoplasmic glutathione S-transferase (cGST)-like domain including a glutaredoxin-like domain. The glutaredoxin-like domain of *Se-mPGES2* had a consensus homology sequence of Cys112-x-x-Cys115 (yellow-colored box in **Figures 1C,D**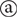). The conserved GSH-binding motif among species was also present in *Se-mPGES2* (blue-colored box in **Figures 1C,D**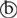). Based on a human mPGES-1 model (Sjögren et al., 2013), this domain may catalyze the isomerization of PGH_2_ to PGE_2_ from the sequence alignment analysis on the conserved amino acid residues (**Figure 1E**).

### Expression Profile of Se-mPGES2

**FIGURE 2:**
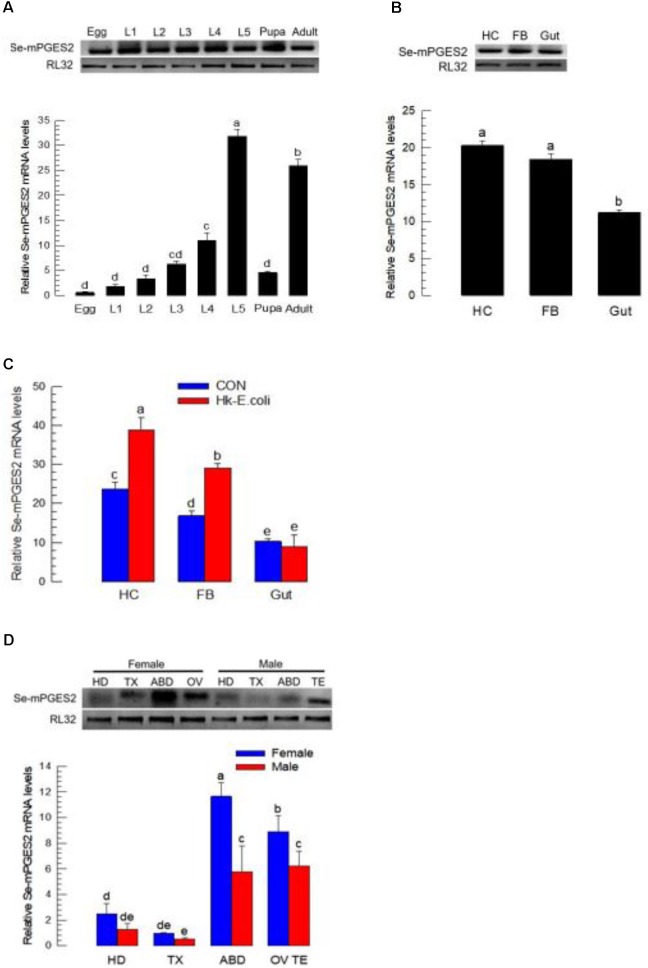
Expression profile of *Se-mPGES2*. **(A)** Expression patterns in different developmental stages: egg, first to fifth instar larvae (“L1–L5”), pupa, and adult. **(B)** Expression patterns in indicated tissues of L5 larvae: hemocyte (“HC”), fat body (“FB”), and midgut (“Gut”). **(C)** Induction of *Se-mPGES2* in response to bacterial challenge. Heat-killed *E. coli* was injected into L5 larvae and incubated for 12 h at 25°C. **(D)** Expression patterns in indicated body parts of adults: head (“HD”), thorax (“TX”), abdomen (“ABD”), ovary (“OV”), and testis (“TE”). A ribosomal gene, RL32, was used as reference gene. Each treatment was replicated three times with independent tissue preparations. Different letters indicate significant differences among means at Type I error = 0.05 (LSD test).

Expression of *Se-mPGES2* was analyzed under selected physiological conditions (**Figure 2**). *Se-mPGES2* was expressed in all developmental stages from egg to adult, with high expression levels during the L5 and adult stages (**Figure 2A**). Selected larval tissues were isolated and assessed for *Se-mPGES2* expression levels (**Figure 2B**). Both immune-associated tissues, hemocytes and fat body, exhibited higher expression levels than the midgut. The expression levels in the immune-associated tissues, but not midguts, were significantly up-regulated following immune challenge (**Figure 2C**). The abdominal region containing reproductive organs, ovaries and testes, exhibited high expression of *Se-mPGES2*, with females higher than males (**Figure 2D**).

### Physiological Role of Se-mPGES2 in Immunity

**FIGURE 3:**
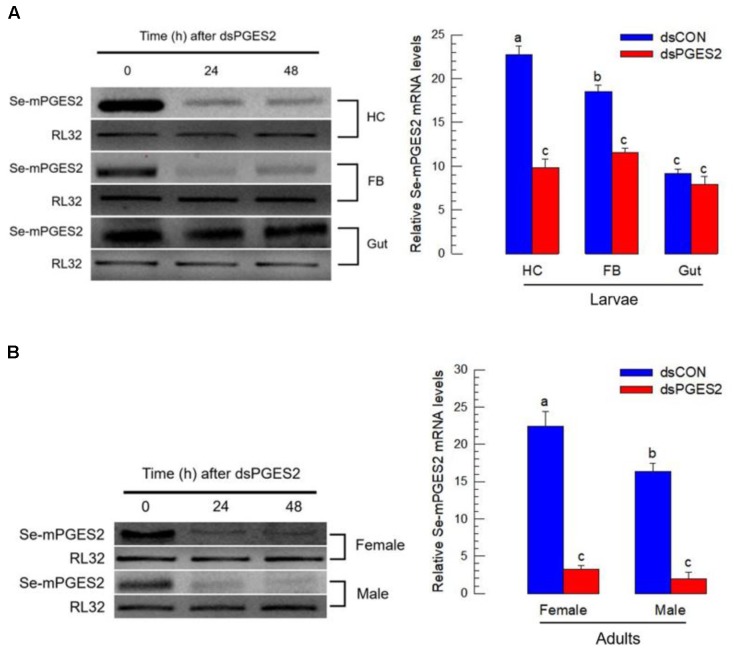
RNA interference (RNAi) of *Se-mPGES2* in larvae and adults. One mg of gene-specific dsRNA (“dsPGES2”) was injected into L5D1 or 5 days old pupae. A viral gene, *CpBV302*, was used as a control dsRNA (“dsCON”). **(A)** Effect of RNAi on *Se-mPGES2* expression in indicated tissues of L5 larvae: hemocyte (“HC”), fat body (“FB”) and midgut (“Gut”). **(B)** Effect of RNAi on *Se-mPGES2* expression in male and female adults. Each treatment was independently replicated three times. Different letters indicate significant differences among means at Type I error = 0.05 (LSD test).

dsRNA treatments led to significant reduction in *Se-mPGES2* expression in hemocytes and fat body, but not midgut, 24 and 48 h PI (**Figure 3**).

**FIGURE 4:**
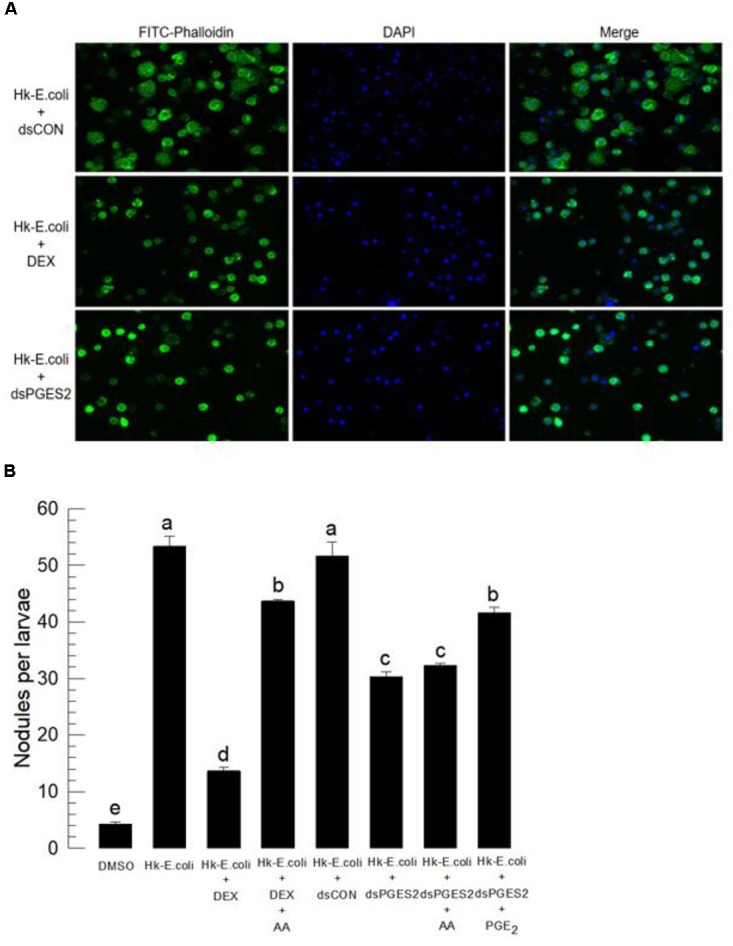
Bioassay of a physiological role of *Se-mPGES2* in cellular immunity after dsRNA treatments, performed as described in **Figure 3**. For a bacterial challenge, heat-killed (HK) *E. coli* (5.4 × 10^4^ cells per larva) was injected into larvae at 48 h after dsRNA treatment. **(A)** Inhibitory effect of dsSe-mPGES2 against F-actin growth in response to bacterial challenge. At 4 h PI, hemocytes were observed under a fluorescence microscope at 40× magnification. Hemocytic F-actin filaments were specifically recognized by FITC-tagged phalloidin (green) while nucleus was stained with DAPI (blue). A PLA_2_ inhibitor, dexamethasone (“DEX”), was used along with bacteria. **(B)** Inhibitory effect of dsSe-mPGES2 against hemocyte nodule formation in response to the bacterial challenge. At 8 h PI, numbers of nodules were assessed. Each treatment was independently replicated five times. Different letters indicate significant differences among means at Type I error = 0.05 (LSD test).

Bacterial challenge stimulated the hemocyte-spreading behavior in control larvae, recorded as extending cytoplasm along with the growth of F-actin. Treating larvae with DEX, an inhibitor of eicosanoid biosynthesis, then with a standard immune challenge, blocked the hemocyte spreading reaction (**Figure 4A**). We recorded similar results with hemocytes prepared from larvae treated with dsRNA specific to *Se-mPGES2*.

We assessed the influence of inhibiting eicosanoid biosynthesis and of silencing *Se-mPGES2* on the hemocyte nodule formation reaction to bacterial challenge (**Figure 4B**). Injection of heat-killed bacteria induced about 53 nodules in control larvae, which was reduced in larvae treated with dexamethasone (DEX). Injecting AA (a PUFA precursor to eicosanoid biosynthesis, into DEX-treated larvae significantly recovered the cellular immune response. Larvae treated with dsRNA specific to *Se-mPGES2* also were significantly impaired in nodule formation. PGE_2_, but not AA, treatments led to recovery of the inhibited immune responses.

**FIGURE 5:**
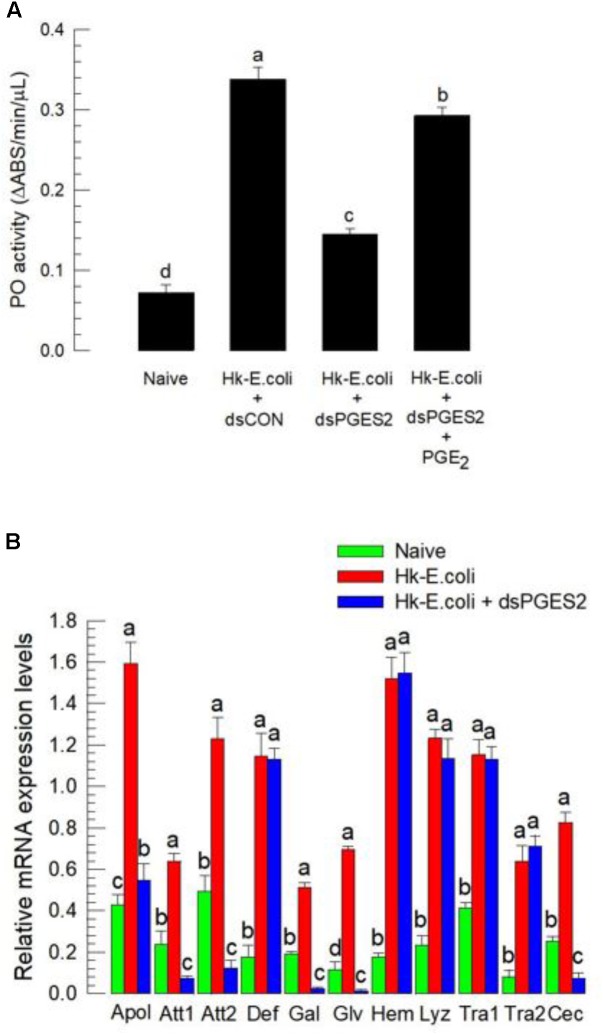
The influence of dsSe-mPGES2 treatments on humoral immunity. dsRNA injected as described in **Figure 3**. For a bacterial challenge, heat-killed (HK) *E. coli* (5.4 × 10^4^ cells per larva) was injected into larvae at 48 h after dsRNA treatment. **(A)** Inhibitory effect of dsSe-mPGES2 on phenoloxidase (PO) activity in response to bacterial challenge. At 8 h PI of the bacteria, PO activity was measured. PGE_2_ (1 μg per larva) was injected along with the bacteria. **(B)** Inhibitory effect of *Se-mPGES2* RNAi against the expression of 11 antimicrobial peptide (AMP) genes: apolipophorin III (“Apol”), attacin 1 (“Att1”), attacin 2 (“Att2”), defensin (“Def”), gallerimycin (“Gal”), gloverin (“Glv”), hemolin (“Hem”), lysozyme (“Lyz”), transferrin 1 (“Tra1”), transferrin 2 (“Tra2”), and cecropin (“Cec”). RL32 was used as an internal control. Each treatment was replicated three times. Different letters indicate significant differences among means at Type I error = 0.05 (LSD test).

Melanization induced by the catalytic activity of PO is essential for both cellular and humoral immune responses in insects (Cerenius and Söderhäll, 2004). PO activity was significantly increased in experimental larvae following injection of heat-killed bacteria (**Figure 5A**). Again, RNAi treatments significantly reduced the enzyme activity, which was rescued by PGE_2_ treatment. Expression of 11 AMP/immune-associated peptide genes, a substantial part of humoral immunity, was up-regulated in response to bacterial challenge (**Figure 5B**). The RNAi treatments suppressed the up-regulation of six of the 11 genes.

### Se-mPGES2 Acts in Reproduction

**FIGURE 6:**
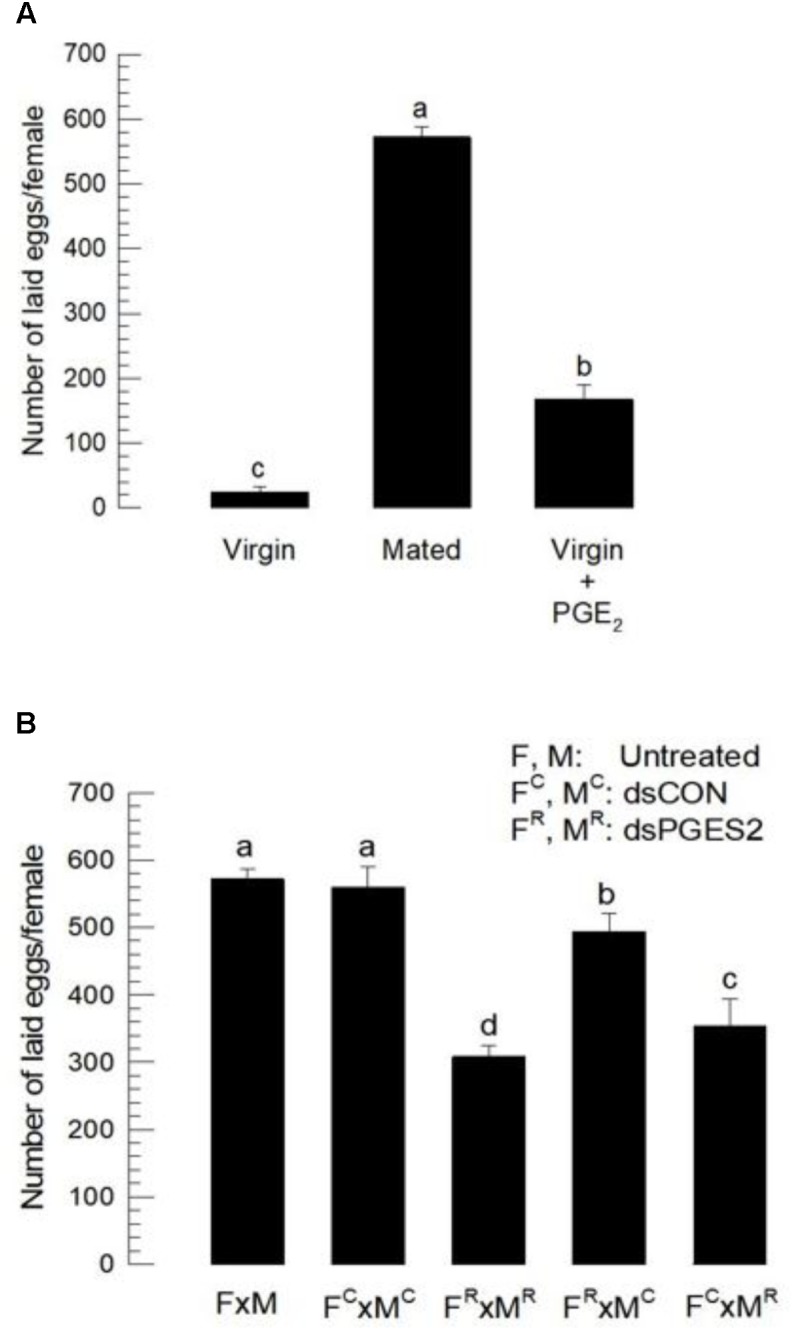
The influence of dsSe-mPGES2 treatments on reproduction. dsRNA was injected as described in **Figure 3** into 5 days old male (“M”) or female (“F”) pupae. A viral gene, *CpBV302*, was used as a control dsRNA (“dsCON”). **(A)** Stimulatory effect of PGE_2_ on egg-laying behavior. Total number of laid eggs by virgin (without male) or mated (with males in 1:1 sex ratio) was counted for 3 days after adult emergence. PGE_2_ (10 μg per adult) was injected to virgin females at the day of adult emergence. **(B)** Inhibitory effect of dsSe-mPGES2 treatments on egg-laying behavior. Each treatment consisted of three replicates. Each replicate used 10 females and/or 10 males. Different letters indicate significant differences among means at Type I error = 0.05 (LSD test).

We tested the hypothesis that PGE_2_ acts in *S. exigua* egg-laying behavior by suppressing *Se-mPGES2* expression with RNAi treatments (**Figure 3B**). After dsRNA injection, the expression of *Se-mPGES2* was markedly reduced in both male and female compared to controls. Mating stimulated oviposition (**Figure 6A**) because mated females laid about 600 eggs while virgins laid less than 20. Injection of PGE_2_ into virgins significantly stimulated egg-laying behavior (**Figure 6B**). Mating between RNAi-treated males and RNAi-treated females (F^R^ × M^R^) significantly reduced the numbers of laid eggs. Mating between RNA-treated female and control male (F^R^ × M^C^) also slightly reduced the egg-laying behavior. There was a much larger reduction in egg-laying following mating between control females and RNA-treated males.

## Discussion

The data reported in this paper strongly support our hypothesis that *Se-mPGES2* mediates biosynthesis of physiologically active PGE_2_ through its cognate protein. Several points are germane. First, we identified a gene encoding a *Se-mPGES2* in a *S. exigua* transcriptome. The gene encodes a protein that is very similar in calculated 3-D structure to a mammalian PGES2. The *S. exigua* gene clusters with other mPGES2s. Second, the gene is expressed in all life stages, particularly in immunity-conferring tissues, fat body and hemocytes, and reproductive tissues, ovaries and testes. Third, *Se-mPGES2* expression increased by nearly twofold following challenged with heat killed bacteria. Fourth, dsSe-mPGES2 injection treatments led to reduced *Se-mPGES2* expression in hemocytes and fat body, but not in gut preparations. Fifth, dsSe-mPGES2 treatments led to suppression of three immunological reactions to bacterial challenge, reduced hemocyte spreading, hemocytic nodulation reactions, and expression of genes encoding AMPs. Finally, dsSe-mPGES2 treatments led to substantially reduced egg deposition. Taken together, these points reveal a key step in PGE_2_ biosynthesis and document crucial PGE_2_ actions in insect biology.

More than 30 mPGES2-like sequences have been identified in insects and other arthropod genomes (Hansen et al., 2014). Two mPGES2 proteins purified from amphipod crustaceans, *Gammarus* sp. and *Caprella* sp. specifically isomerize PGH_2_ into PGE_2_ (Hansen et al., 2014). *Se-mPGES2* exhibited high sequence homologies (90–95%) with the amphipod PGES2s, from which we infer that *Se-mPGES2* also catalyzes the isomerization reaction. This is strongly supported by our functional assays reported in this paper.

Knockdown of *Se-mPGES2* expression led to suppression of three immune functions in last instar *S. exigua* larvae. dsSe-mPGES2 treatments led to significantly reduced nodule formation compared to control larvae, which was reversed by subsequent PGE_2_, but not AA, treatments. AA treatments reverse the influence of DEX treatments on nodulation and on hemocyte migration in other insect species (Stanley and Kim, 2011, 2014), raising the question of why the AA treatments did not reverse the influence of dsSe-mPGES2? We infer that the AA injected into experimental larvae was converted into PGH_2_, but under dsSe-mPGES2 treatment, the PGH_2_ could not be converted into PGE_2_. PGE_2_ mediates mobilization of insect hemocytes from sessile to circulatory forms (Park et al., 2014), which increases hemocyte populations to effectively defend against invaders. PGE_2_ also stimulates hemocyte-spreading behavior to facilitate cellular immune responses including nodule formation (Srikanth et al., 2011). PGE_2_ induces prophenoloxidase (PPO) release from oenocytoid hemocytes by cell lysis (Shrestha and Kim, 2008). The released PPO is then activated to phenoloxidase (Jiang et al., 2009), which acts in the melanization step of nodule formation. *Se-mPGES2* is required for nodule formation of *S. exigua* in response to bacterial infection. DEX treatments also inhibited another cellular immune function, cell spreading, seen in the influence on cytoskeleton functions. Cell spreading is a key immune function in nodulation and in wound responses.

dsSe-mPGES2 treatments suppressed *S. exigua* humoral immune responses. Eicosanoids mediate humoral immune response by blocking expression of genes encoding AMPs (Kim et al., 2018). Similarly, inhibiting eicosanoid biosynthesis in *B. mori* led to reduced expression of genes encoding lysozyme and the AMP, cecropin (Morishima et al., 1997). Yajima et al. (2003) demonstrated a functional link between eicosanoid biosynthesis and the IMD signal pathway, which regulates expression of genes encoding AMPs in *D. melanogaster*. The Toll signal pathway is associated with eicosanoid biosynthesis in *S. exigua* (Shafeeq et al., 2018). PGA_1_, PGE_1_, and PGA_2_ treatments led to altered gene expression in an insect cell line derived from *Helicoverpa zea* (Stanley et al., 2008, 2012). The linkages between PGE_2_ and the signal pathways responsible for regulating expression of AMP-encoding genes highlights the *Se-mPGES2* actions necessary to produce immune signaling PGE_2_.

Aside from expression and actions in immunity, *Se-mPGES2* is substantially expressed in adults, particularly in ovaries and testes. PGs act is several aspects of reproductive biology in invertebrates and vertebrates (Stanley, 2000). Particularly in female insects, PGE_2_ acts in several aspects of ovarian development (Tootle, 2013). mPGES2 of *D. melanogaster* acts in male fly fertility (Bichon et al., 2001). Our data show that mating between untreated males and females and between males and females treated with a control dsRNA construct led to deposition of nearly 600 eggs per female. Mating between dsSe-mPGES2-treated males and females led to far reduced egg laying, down by about 50%. Mating between dsSe-mPGES2-treated females and control males led to a small decrease in egg laying. Similar mating between control females and dsSe-mPGES2-treated males led to large drop in egg laying, down from about 600 eggs/female to about 380 eggs/female. We infer that PGE_2_ mediates egg laying in *S. exigua*.

Because mating with dsSe-mPGES2-treated males led to reduced egg laying, we draw on Loher (1979) and Loher et al. (1981) to suggest a possible mechanism of the PGE_2_ action in *S. exigua*. Males of the Australian field cricket, *Teleogryllus commodus*, transfer sperm and other seminal fluid components into spermathecae of females via a spermatophore. Successful mating leads to deposition of 100s of fertilized eggs into the mating arena substrate over the first several hours after mating. Loher (1979) reported that injections of PGE_2_ into virgin, gravid females, similarly, promoted deposition of large numbers of sterile eggs. Later biochemical work showed that spermathecae from mated females contained about 500 pg of PGE_2_ per spermatheca while spermathecae from virgins contained no detectable PGE_2_. Spermatophores contained about 20 pg PGE_2_ per spermatophore, far less than the 500 pg found in spermathecae. Investigation of PGE_2_ biosynthesis showed that spermatophores biosynthesize PGE_2_ at about 25 pmol/h/spermatophore, not far different from the approximately 35 pmol/h/spermatheca recorded from spermathecae prepared from mated females. Spermathecae from virgins had negligible PG synthesizing activity. These findings gave rise to the “enzyme transfer model,” in which a PGE_2_ synthase is transferred to females during mating, which forms PGs within spermathecae. The PGs move into circulating hemolymph, where they release the egg-laying behavioral program, located in the terminal abdominal ganglion. In later work Stanley-Samuelson et al. (1987) injected radioactive AA into male crickets. They detected the labeled AA in spermatophores for the following 49 days. Some of the radioactivity was recovered in spermathecae and in hemolymph of females that had mated with the radioactive males. Characterization showed that some of the hemolymph radioactivity was present in the forms of PGA_2_, PGE_2_, and PGF_2α_. Many lepidopterans mate by spermatophore transfer. Because dsSe-mPGES2 treatments in males led to reduced egg-laying in females, we hypothesize that males transfer PGs *per se* in their seminal fluids, which releases egg-laying behavior in females.

We identified a PGE_2_-synthesizing enzyme and two of its biological functions in *S. exigua*. PG biosynthetic pathways have been unclear in insects due to lacking information on enzymes orthologous to those of vertebrates (Kim et al., 2018). The identification of *Se-mPGES2* is a major step toward understanding biosynthesis of one PG, PGE_2_, in insects. PGH_2_ is the direct precursor to several primary PGs, PGA, PGB, PGD, PGE, PGF, thromboxane, and prostacyclin (or PGI; Stanley, 2000). Each PG is produced by cell specific enzymes and, with the exception of PGES2, none of these have been characterized in insect models.

In *S. exigua*, two calcium-independent PLA_2_s have been reported (Park et al., 2015; Sadekuzzaman et al., 2017). These two enzymes act in hydrolysis of PUFAs from cellular phospholipids, the first step in PG biosynthesis. The second step in insects is catalyzed by COX-like peroxinectins, identified in *D. melanogaster* and *S. exigua* (Tootle, 2013; Park et al., 2014, 2015). Here, we report on the third and final step, isomerization of PGH_2_ to PGE_2_ by *Se-mPGES2.* Yamamoto et al. (2013a,b) reported the active site of the *B. mori* mPGES2 features an electron-sharing network at Asn95, Asp96, and Arg98. *Se-mPGES2* is highly homologous to the amphipod and silkworm PGES2s in predicted amino acid sequences, in which a GSH binding motif (Cys-x-x-Cys) and the electron-sharing network are conserved. We infer *Se-mPGES2* is responsible for PGE_2_ biosynthesis in *S. exigua*.

## Author Contributions

YK conceived and designed the experimental plan. SA performed the experiments and drafted the manuscript. YK and SA analyzed the data. YK and DS refined and approved the final manuscript.

## Conflict of Interest Statement

The authors declare that the research was conducted in the absence of any commercial or financial relationships that could be construed as a potential conflict of interest.

## Footnotes

1 http://www.ncbi.nlm.nih.gov

2 https://www.ebi.ac.uk/Tools/msa/clustalw2/

3 http://pfam.xfam.org

4 https://prosite.expasy.org/

5 https://spdbv.vital-it.ch/

6 https://www.cgl.ucsf.edu/chimera/
