# Supplementary material for: An Insect Prostaglandin E2 Synthase Acts in Immunity and Reproduction
Source: Front Physiol. 2018 Sep 4;9:1231. doi: 10.3389/fphys.2018.01231 (PMC6131586; doi:10.3389/fphys.2018.01231)
Supplement: Supplementary file 1 [file Table_1.DOCX]

**Table S1.** Primers used in this study

| Genes | Primer sequences |
| --- | --- |
| *Se-mPGES2* | 5´-GAG TCC TGG TCA ACA CAC TAT C-3´  5´-CAC TGT CAC GTC GGC TAA A-3´ |
| Apolipophorin III | 5´-AGT GTC GCC AAG TTG TTC GTG-3´  5´-CTC CTG CGC GGT GTT CTG CA-3´ |
| Attacin 1 | 5´-GCT TTC CTC TCC AGG AAT ATG-3´  5´-CCT TAG AGT AAA TCC AGT GG-3´ |
| Attacin 2 | 5´-TCC CGA ATG TGC CCA ACT TC-3´  5´-GAA AGA TCT GCC GAA AGT AAG-3´ |
| Defensin | 5´-ATG GGT GTT AAG GTA ATA AAT GTG-3´  5´-GCA ACT ACA TGT ATG ACT AAC GC-3´ |
| Gallerimycin | 5´-TCA GTC ATG AAA GCT TGC GTA-3´  5´-TCG CAC ACA TTG GCA TCC ATT C-3´ |
| Gloverin | 5´-CGT GGA CAT CTT CAG GGC C-3´  5´-GTC GTG TTC AAT GCC ACC-3´ |
| Hemolin | 5´-AAG ACC AGG GCG AGT ACA AG-3´  5´-AGC GAC ATG AAC CAA GGT TTC-3´ |
| Lysozyme | 5´-ATG CAA AAG CTA ACG GTT TTC-3´  5´-GAT TCT TCC ATC CAT ACC AG-3´ |
| Transferrin 1 | 5´-GTC CCT CTC TGT CCT GAA GG-3´  5´-CAG AAA CAC GAA GAA AGA TGG-3´ |
| Transferrin 2 | 5´-GAT GTT CTG GCG CAG CTG TC-3´  5´-CCG GCT GAA CGC AAA CAC AG-3´ |
| Cecropin | 5´-ATC GTT TAG CTT CGT GTT CGC-3´  5´-CTT TCT TTT ACC ACA CGG TTG-3´ |
